# Lipopolysaccharide upregulates miR-132/212 in Hirschsprung-associated enterocolitis, facilitating pyroptosis by activating NLRP3 inflammasome via targeting Sirtuin 1 (SIRT1)

**DOI:** 10.18632/aging.103852

**Published:** 2020-09-20

**Authors:** Hongxing Li, Lingling Zhou, Zhengke Zhi, Xiurui Lv, Zhonghong Wei, Xin Zhang, Weibing Tang, Meiling Tong

**Affiliations:** 1 Department of Neonatal Surgery, Children’s Hospital of Nanjing Medical University, Nanjing, China; 2 Department of Pediatrics, Nanjing Medical University, Nanjing, China; 3 Department of Pediatrics, Women’s Hospital of Nanjing Medical University, Nanjing Maternity and Child Health Care Hospital, Nanjing, China

**Keywords:** Hirschsprung-associated enterocolitis, lipopolysaccharide, miRNA, NLRP3, pyroptosis

## Abstract

Hirschsprung disease (HSCR) is a congenital disorder attributed to the failure of the neural crest derivatives migrating and/or differentiating along the hindgut. The most frequent complication in Hirschsprung disease patients is Hirschsprung-associated enterocolitis (HAEC). However, its pathogenesis has not been fully understood. This study investigated miRNAs influenced by Lipopolysaccharide (LPS) in postoperative HAEC patients, their effect on enterocolitis and the underlying mechanism. MiR-132 and miR-212 were up-regulated in HAEC dilated tissues and LPS-treated mice enteritis samples. LPS-stimulated HT29 cells showed a high expression of miR-132 and miR-212. QRT-PCR analysis, western blotting, luciferase reporter assay, and flow cytometric analysis were carried out in vitro, showing that miR-132 and miR-212 could directly inhibit Sirtuin 1 (SIRT1) expression. Consequently, SIRT1 deficiency in LPS-stimulated HT29 cell line and LPS-treated mice activated NLRP3 inflammasome and Caspase-1-mediated pyroptosis. Furthermore, the above inflammation activation was reversed by miR-132/212 inhibitor or SIRT1 overexpression plasmid transfection.

In conclusion, LPS upregulated miR-132 and miR-212 expression in HAEC, suppressing SIRT1 and facilitating NLRP3 inflammasome activation, which induced pyroptosis. Our findings illustrated the role of LPS/miR-132/-212/SIRT1/NLRP3 regulatory network in the occurrence and progression of HAEC and proposed a new molecular pathway for LPS-mediated cell pyroptosis.

## INTRODUCTION

Hirschsprung disease (HSCR) is a neonatal neurocristopathy arising from a defect of enteric neurons in the distal colon, with an incidence of 1 in 5000 live births (live born infants) [1, 2]. Clinical manifestations of HSCR patients usually include constipation, vomiting, abdominal distension and feeding difficulty [3]. In spite of recent advances in invasive surgery to remove or bypass aganglionic bowel sections to deal with HSCR, risk of Hirschsprung-associated enterocolitis(HAEC) with severe morbidity remains in these patients [4]. HAEC is a life-threatening syndrome of HSCR, which can pose a threat to patients preoperatively or postoperatively [5]. Due to the assistance of rectal decompression and antibiotic therapy, we now have little chance to encounter the typical clinical manifestations of preoperative HAEC in clinics. However, postoperative HAEC still has an incidence of 35% [6, 7].

Despite the poor understanding of the pathophysiology of HAEC, several recent studies have shed light on the details of HAEC development. Alterations in innate immune responses, impaired gastrointestinal mucosal barrier function, anomalous intestinal microbiome and bacterial translocation may account for the mechanism of HAEC [8]. It has been reported that the bacterial composition of patients with HAEC was characterized by elevated gram-negative bacteria in comparison with the HSCR group [9–11]. Our previous study has also found that gram-negative bacteria-released LPS is prominently increased in postoperative HAEC patients [12], suggesting that gut microbiota dysbiosis and subsequently released LPS appeared to be important elements of HAEC.

The formation of excessive inflammatory response may be the key to the occurrence and further deterioration of HAEC. Interestingly, LPS can mediate cell pyroptosis through a non-classical cell pyroptosis pathway, which plays an important role in intestinal inflammation [13]. What’s more, LPS can also indirectly activate NLRP3-Caspase-1 inflammasome and release active pro-inflammatory cytokines (IL-1β and IL-18) [14]. However, how does LPS mediate cell inflammation in HAEC? Recent evidence indicated that the expression of miR-132/-212 was significantly elevated in LPS-treated macrophages and human bronchial epithelial cells [15, 16], and LPS can also participate in the inflammatory response by regulating miR-132/-212 [17]. Interestingly, Sirtuin 1 (SIRT1), the common target gene of miR-132 and miR-212, is closely related to cell pyroptosis and inflammation [18]. Therefore, this study innovatively proposed a new molecular pathway in which LPS mediates pyroptosis indirectly, and aimed at defining the pathophysiological role of LPS/miR-132/-212/SIRT1 in HAEC.

Herein, we reported that miR-132/-212 was markedly increased in patients with postoperative HAEC and experimental animal models and discovered the ability of LPS to promote miR-132/-212 expression. Moreover, miR-132/-212 facilitated activation of NLRP3 inflammasomes by down-regulating the expression of SIRT1 in HT29 cell line. Our findings provided information on the pathogenesis of HAEC and may propose a new molecular pathway for LPS-mediated cell pyroptosis.

## RESULTS

### Clinical information analysis

This study cohort comprised 32 HSCR cases and 32 postoperative HAEC patients. Detailed demographic and clinical inflammation of patients were listed in Table 1, including age, sex, diet and surgical history. No statistically significant differences were observed between 32 HSCR cases (No-HAEC) and 32 postoperative HAEC groups (Po-HAEC).

**Table 1:** Clinical characteristics of study population.

**Variable**	**No-HAEC (n=32)**	**Po-HAEC (n=32)**	**P**
**Age at surgery(days, mean, SE)**	122.2 (9.807)	123.0 (7.556)	0.948^a^
**Sex (%)**			
Male	30 (93.75)	28 (87.5)	0.391^b^
Female	2 (6.25)	4 (12.5)	
**^c^Diet (%)**			
Exclusive breastfeeding	8 (25)	14(43.75)	0.104^b^
Mixed feeding	16 (50)	8 (25)	
Artificial feeding	8 (25)	10 (31.25)	
**^d^LSS**			
YES	14	20	0.133^b^
NO	18	12	
**Fistulation**			
YES	2	0	0.151^b^
NO	30	32	
**HSCR classification**			
Short-segment HSCR	19	20	0.599^b^
Long-segment HSCR	12	12	
Total colonic aganglionosis HSCR	1	0	

^a^ Student’s t-test.

^b^ Two-sided chi-squared test.

^c^Diet: Diet in the first 6 months of life

^d^LSS: Laparoscopic soave surgery

### Upregulation of miR-132/-212 in patients with postoperative HAEC and enteritis animal models

**Figure 1:**
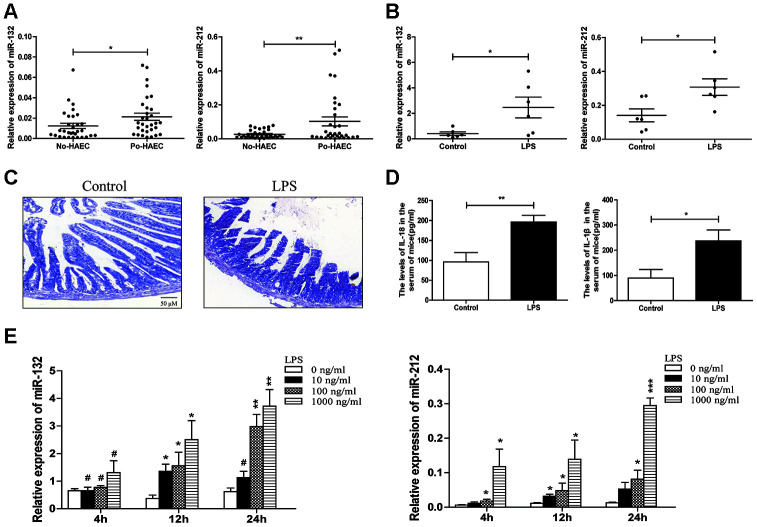
**Upregulation of miR-132/-212 in postoperative HAEC patients or enteritis animal models.** (**A**) The expression of miR-132/212 was validated in dilated segments of 32 po-HAEC and 32 No-HAEC patients using qRT-PCR. MiR-132 and miR-212 expression were significantly increased in po-HAEC tissues comparing to No-HAEC samples. (**B**) qRT-PCR was applied to detect miR-132 and miR-212 expression in LPS-stimulated mice and control groups (n=6 each). (**C**) The pathological changes in mice challenged with LPS was observed by HE staining. Scale bar: 50 μM. (**D**) Mean serum concentrations of IL-18 and IL-1β in LPS-stimulated mice and control groups was evaluated using ELISA assay. Data were expressed as mean ± SD. *P<0.05, ** P<0.01. (**E**) qRT-PCR analysis was applied to detect dose- and time-dependent expression of miR-132 and miR-212 in HT29 cell line treated with 0-1000 ng/ml LPS for 4-24 h.

The relative miR-132 and miR-212 levels were measured among dilated segments of 32 Po-HAEC and 32 No-HAEC patients by qRT-PCR. The results verified the marked overexpression of miR-132/-212 in Po-HAEC patients (Figure 1A). In line with the above findings, the expression of miR-132 and miR-212 was notably upregulated in intestinal tissues of animal models treated with LPS (Figure 1B). Morphological changes were inspected in control mice and experimental enteritis mice. We observed partial loss of villi with separation in the submucosa and lamina propria in HE staining of mice challenged with LPS. Meanwhile no histologic abnormalities were shown in the control group (Figure 1C). Consistently, the serum concentrations of pro-inflammatory cytokines (IL-18 and IL-1β) were significantly increased in LPS-induced mice group (Figure 1D), indicating the successful establishment of the enteritis model. To further verify the relationship between LPS and high expression of miR-132/-212, HT29 cells were used for vitro experiments. LPS-stimulated HT29 cells showed rapid and dramatic increase of miR-132 and miR-212 (Figure 1E). We primarily opted for the condition of 100ng/ml and 1000 ng/ml LPS treatment (for 24 hours) in subsequent experiments. Collectively, the above data indicated that miR-132 and miR-212 levels were substantially over-expressed in HAEC.

### SIRT1 dysregulation by LPS as a result of miR-132 and miR-212 overexpression

**Figure 2:**
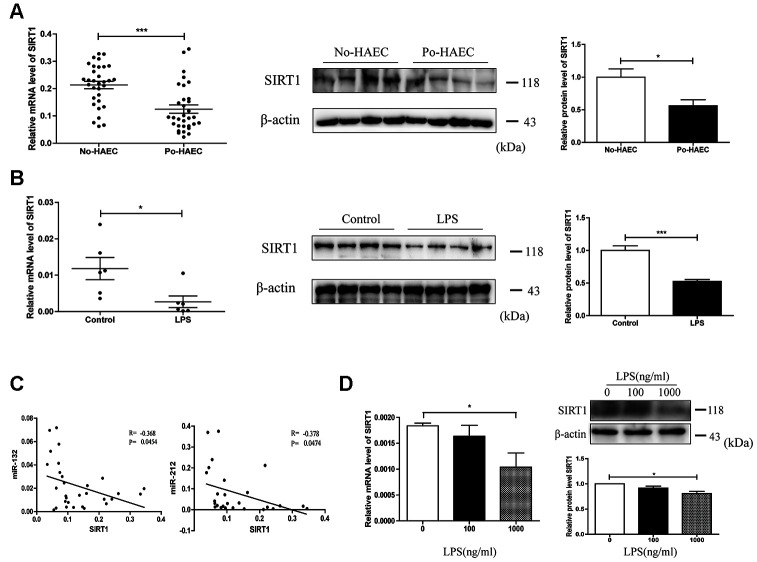
**SIRT1 dysregulation by LPS as a result of miR-132 and miR-212 overexpression.** (**A**) SIRT1 expression was tested to be down-regulated in dilated segments of 32 po-HAEC compared with 32 No-HAEC patients by qRT-PCR. As revealed by western blot analysis, SIRT1 protein level was declined in 4 pairs of po-HAEC tissues compared to No-HAEC samples. (**B**) In comparison with control mice group, SIRT1 mRNA level was decreased in LPS-stimulated mice intestinal tissues. The protein expression of SIRT1 was notably decreased in animal models treated with LPS. (**C**) SIRT1 was inversely correlated with miR-132 and miR-212 levels in the same paired dilated tissues (P=0.0454, P=0.0474, Pearson). (**D**) Both mRNA and protein levels of SIRT1 were down-regulated in HT29 cell line after treatment with LPS for 24h. Mean ± SD. *n* = 3, *P < 0.05.

To investigate how miR-132/-212 played a role in the progress of HAEC, we focused on the target gene of miR-132/-212 that is significantly correlated with inflammation activation processes by using bioinformatic prediction (TargetScan, miRanda, PicTar, DIANA). It was determined that SIRT1 (Sirtuin 1), which has been reported to act as the upstream regulatory factors of the NLRP3 inflammasome, may participate in HAEC as a potential target gene of miR-132/-212 [19]. Then qRT-PCR was conducted to measure the mRNA level of SIRT1 in dilated segments of patients with or without postoperative HAEC. As shown in Figure 2A left, SIRT1 expression was significantly lower than that of No-HAEC tissues. Similarly, there was a decline in SIRT1 expression in intestinal tissues of LPS-stimulated mice compared with that of controls (Figure 2B left). SIRT1 protein expression was in parallel with the mRNA levels (Figure 2A right and 2B right). In an effort to find the correlation between increase of miR-132 and miR-212 and the decrease in SIRT1 level, bivariate correlation analysis was performed. The results showed that miR-132 and miR-212 expression were inversely correlated with SIRT1 mRNA level in the same paired intestinal samples (Figure 2C). We then wondered if LPS-stimulated HT29 cells showed a continuous change of SIRT1 expression in vitro. In HT29 cells incubated with 100 or 1000ng/ml LPS, the expression of SIRT1 was significantly diminished (Figure 2D).

### MiR-132 and miR-212 modulates the function of SIRT1

**Figure 3:**
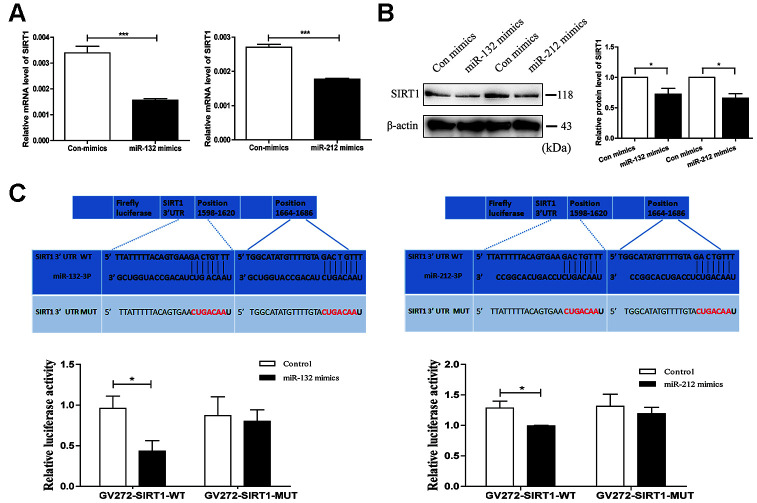
**SIRT1 is a direct target gene of miR-132 and 212.** (**A** and **B**) qRT-PCR and western blot analysis validated respectively mRNA and protein levels of SIRT1 after transfection with miR-132/212 mimics (or Con mimics) in HT29 cell line. Mean ± SD. *n* = 3, *P < 0.05. (**C**) The binding sites within wild type or mutant SIRT1 3’UTR and miR-132 and miR-212. The luciferase activity of wild type or mutant SIRT1 3’UTR after transfection with miR-132 or miR-212 mimics was assessed by dual luciferase reporter assay in HT29 cell line. Con, control.

To further study whether miR-132 and miR-212 interacted with SIRT1, we transfected miR-132 and miR-212 mimics in HT29 cell line. qRT-PCR analysis was carried out to evaluate miR-132/-212 expression levels in HT29 cell line treated with miRNA mimics (Supplementary Figure 1A). Then, the following results verified the negative influence of miR-132/-212 to SIRT1 mRNA and protein levels (Figure 3A and 3B). In view of the nearly perfect pairing between the SIRT1-3′untranslated region (UTR) and the miR-132/-212 seed sequence (Figure 3C upper), in-vitro assays were then used to confirm the ability of miR-132 and miR-212 to suppress SIRT1. The dual luciferase assay indicated that relative-luciferase vitality was inhibited in HT29 cells transfected with miR-132 and miR-212 mimics and plasmid GV272-SIRT1-Wild. Nevertheless, there was no obvious difference in relative-luciferase vitality when HT29 cells were transfected with GV272-SIRT1-Mut and miR-132/-212 mimics compared with GV272-SIRT1-Mut and Control (Figure 3C lower). In general, the findings proved that SIRT1 was a direct target of miR-132/-212.

### Activation of NLRP3 inflammasome in postoperative HAEC patients and LPS-stimulated HT29 cell line

**Figure 4:**
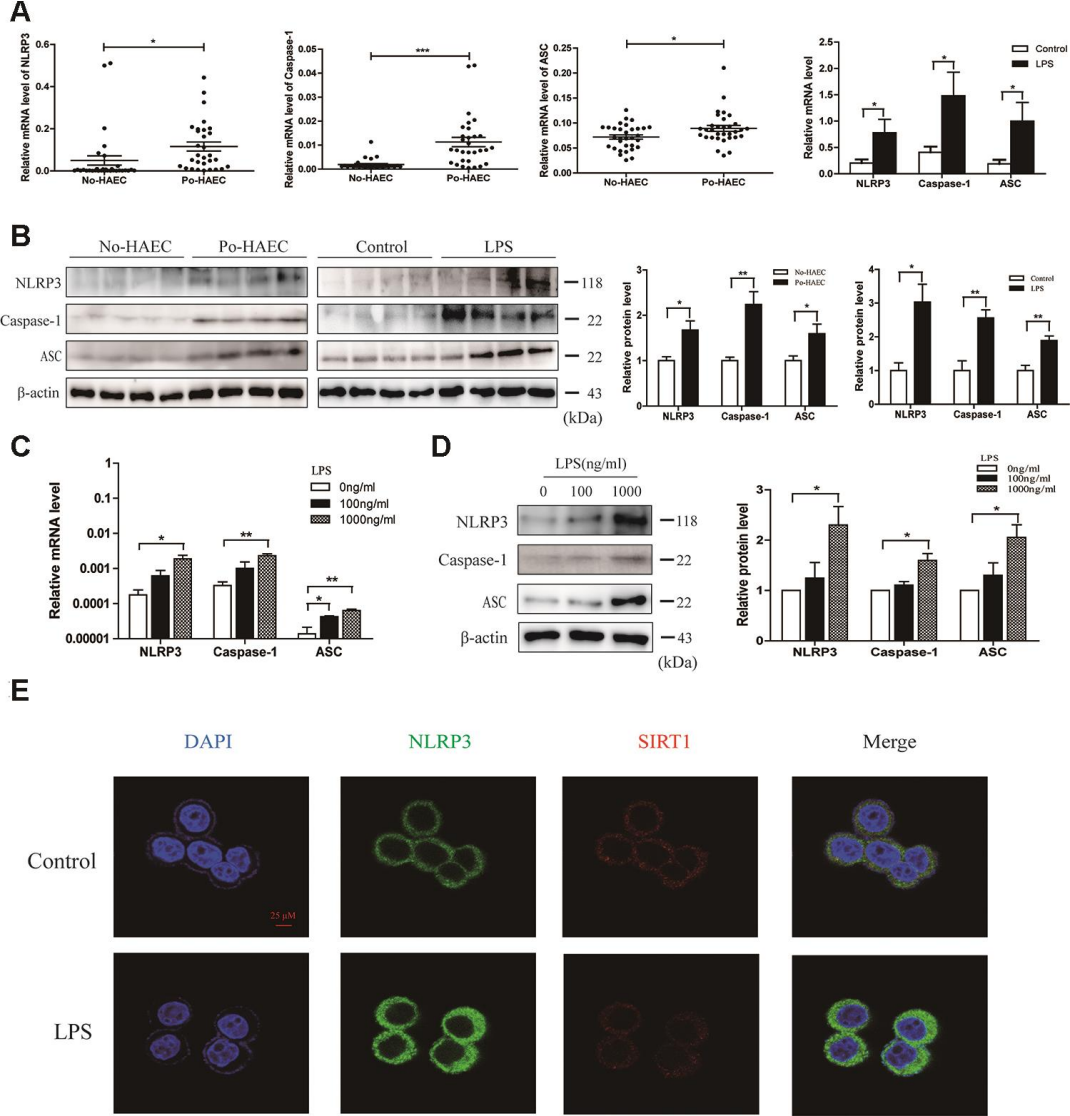
**Activation of NLRP3 inflammasome in patients with postoperative HAEC and LPS-stimulated HT29 cell line.** (**A**) qRT-PCR validated relative mRNA levels of NLRP3, Caspase-1 and ASC in dilated segments of 32 po-HAEC and 32 No-HAEC patients. The above genes mRNA expression was distinctly increased in po-HAEC tissues. Compared with control mice tissues, NLRP3, Caspase-1 and ASC mRNA levels showed a increase in mice intestinal tissues challenged with LPS. (**B**) The protein levels of NLRP3, Caspase-1 and ASC were up-regulated in 4 pairs of po-HAEC tissues compared to No-HAEC samples. LPS treatment to mice increased NLRP3, Caspase-1 and ASC protein expression of intestinal tissues. β-actin was regarded as an internal control. (**C** and **D**) The mRNA and protein levels of NLRP3, Caspase-1 and ASC were upregulated in HT29 cell line after treatment with LPS for 24h. Mean ± SD. *n* = 3, *P < 0.05. (**E**) Confocal images of SIRT1 and NLRP3 in HT29 cell line treated with 1000 ng/ml LPS for 24 h. Scale bar =25μm.

Inflammasome activation has been proved to account for many disease progression [20]. In order to find out whether activation of NLRP3 inflammasome participated in HAEC, the relative NLRP3, ASC and Caspase-1 levels were determined among dilated segments of 32 Po-HAEC and 32 No-HAEC groups by qRT-PCR. As shown in Figure 4A, these genes level showed a significantly increase in po-HAEC group, and so were in mice challenged with LPS. Consistently, similar changes of these genes at protein expression were observed in patients with po-HAEC and LPS-stimulated mice (Figure 4B). To assess whether LPS treatment can induce pro-inflammatory microenvironment, we incubated HT29 cells with 100 or 1000ng/ml LPS. As expected, LPS increased the mRNA and protein levels of NLRP3, ASC and Caspase-1 in vitro (Figure 4C and 4D). Moreover, immunocytochemical assays were employed to evaluate the localization of NLRP3 and SIRT1. NLRP3 staining intensity was enhanced while SIRT1 showed a downward trend upon LPS treatment (Figure 4E). The results of immunofluorescence quantitative analysis were listed in Supplementary Figure 1D.

### Upregulation of miR-132/-212 triggers pyroptosis in HT29 cell line

**Figure 5:**
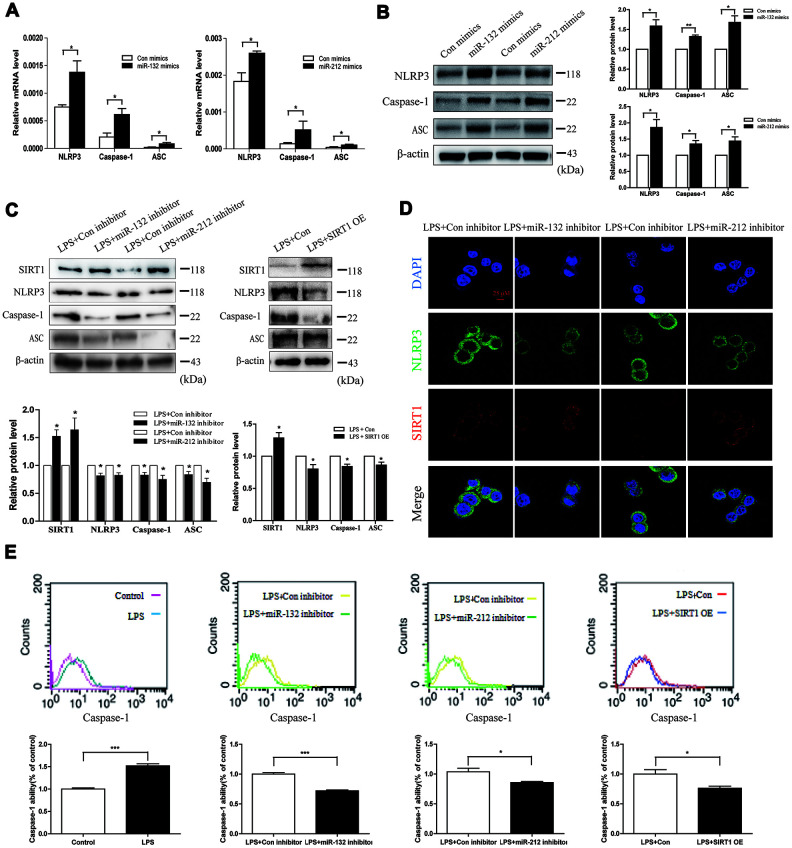
**Upregulation of miR-132/212 triggers pyroptosis in HT29 cells.** (**A** and **B**) qRT-PCR and western blot analysis of NLRP3, Caspase-1 and ASC were detected in HT29 cell line after transfection with miR-132/212 mimics (or Con mimics). Mean ± SD. *n* = 3, *P < 0.05. (**C**) Western blot analysis showed that the protein level of SIRT1 was up-regulated in HT29 cell line exposed to 1000 ng/ml LPS for 24h immediately after transfection with miR-132/212 inhibitor, while NLRP3, Caspase-1 and ASC protein levels were decreased. Data are means ± SEs of three independent experiments. (**D**) Confocal images were performed to determine the staining intensity of SIRT1 and NLRP3 in HT29 cell line treated with 1000 ng/ml LPS for 24h after transfection with miR-132/212 inhibitor (or Con inhibitor). Scale bar =25μm. (**E**) Flow cytometric analysis using active caspase-1 marker was examined in HT29 cell line treated with no or 1000 ng/ml LPS for 24h. MiR-132/212 inhibitor and SIRT1 OE transfection were separately done in HT29 cell line, and then HT29 cell line was exposed to 1000 ng/ml LPS for 24h. Con, control; OE, overexpression.

Since previous research have given evidence of the anti-inflammatory function of SIRT1 via inhibiting NLRP3 inflammasome and IL-1β [21, 22], we focused on the role of miR-132/-212-SIRT1-NLRP3 inflammasome in LPS-induced cell injury. MiR-132 and miR-212 mimics were transfected into HT29 cells, which increased the mRNA and protein expression of NLRP3, ASC and Caspase-1 (Figure 5A and 5B). Meanwhile, we constructed the plasmid to over-express SIRT1 and miRNA inhibitors to suppress the expression of miR-132/-212. Their transfection efficiency was measured by qRT-PCR (Supplementary Figure 1C and 1D). As shown in Figure 5C left, miR-132 and miR-212 inhibitor could partially abrogate LPS promotion effect. Similarly, SIRT1 overexpression resulted in a decrease in the level of active NLRP3, Caspase-1 and ASC (Figure 5C right). MiR-132 and miR-212 inhibitor transfection increased SIRT1 staining intensity and suppressed NLRP3 staining intensity in LPS-stimulated HT29 cells (Figure 5D). We showed the results of immunofluorescence quantitative analysis in Supplementary Figure 1E.

Then we wondered whether LPS elicited HT29 cell pyroptosis considering that Caspase-1 could facilitate pyroptosis. In the flow cytometric analysis using the pyroptosis biomarker (active Caspase-1), we observed that LPS stimulation increased the cell population of high Caspase-1 fluorescence intensity and the mean value of cell Caspase-1 fluorescence intensity, suggesting that LPS augmented Caspase-1 ability in HT29 cells (Figure 5E left), and this influence was cut down by miR-132 and miR-212 inhibitor transfection (Figure 5E middle). SIRT1 overexpression also partially eliminated LPS-stimulated Caspase-1 activation (Figure 5E right). All of the above results verified that upregulation of miR-132/-212 could trigger pyroptosis by activating NLRP3 inflammasome.

## DISCUSSION

Hirschsprung-associated enterocolitis (HAEC) is characterized by explosive diarrhea, caused by a progressive inflammatory response in the small intestine and colon [23]. However, we know little about the underlying basis of the inflammatory response in HAEC. Given that lots of research has been done about diagnosis and treatment of Hirschsprung-associated enterocolitis (HAEC), we tried to explore the pathogenesis for HAEC. Our previous study has found that Gram-negative bacteria-released LPS noteworthily enhanced in HAEC patients prior to diagnosis [12]. It has been proved that LPS facilitates the process of intestinal inflammation [24]. Consistently, our study showed that LPS injection induced enteritis in mice model, as indicated by the characteristic changes in HE staining and release of inflammatory cytokines.

Pyroptosis, also known as inflammatory cell death, is different from apoptosis. It is characterized by mainly relying on Caspase-1, 4, 5, 11 pathway-mediated inflammatory cell necrosis, which is closely related to inflammatory diseases [25]. The pyroptosis pathway is divided into classical and non-classical pyroptosis pathways. The classical pathway is mediated by inflammasome, which mainly depends on Caspase-1, and the non-classical pathway is activated by LPS, which mainly depends on Caspase-4, 5, 11 [26]. Among them, the NLRP3 inflammasome is the most studied. On the other hand, LPS can indirectly activate the NLRP3 inflammasome and activate Caspase-1 to mediate cell pyroptosis [27]. It has been reported that activation of Caspase-1 is responsible for the maturation of pro-IL-1β and pro-IL-18 and secretion of IL-1β and IL-18. Hence, we chose to evaluate the concentrations of pro-inflammatory cytokines (IL-1β and IL-18) in mice model. In this study, we confirmed high expression of Caspase-1 and NLRP3 in dilated segments of postoperative HAEC patients. However, all patients’ dilated segments were collected at the time of surgery when HAEC had not occurred. Thus, IL-1β and IL-18 levels in HAEC may not correctly reflect the inflammation levels of postoperative HAEC patients which may have been compensated by complex organisms.

miR-132 and miR-212 have been verified as LPS-related miRNAs in several cell models [28, 29]. Yang D et al reported that miR-132/-212 played an important role in the process of inflammation-related diseases [30]. Hence, it may lead to crucial findings to focus on whether miR-132/-212 exerts effect on the occurrence and progression of HAEC. In this study, we reported that the expression of miR-132/-212 was increased in HAEC dilated tissue specimens compared with HSCR. In addition, LPS treatment promoted miR-132/-212 levels in mice model and HT29 cell line. MiR-21 was shown to promote the expression of NLRP3, ASC, and Caspase-1 in septic shock [31]. However, miR-132/-212 regulation of NLRP3 inflammasome is still little unknown in the progression of intestinal inflammation, including HAEC.

Previous studies have reported that the sirtuin protein family showed its significance in regulating cell metabolism by its influence on some biological processes in CNS, liver, pancreas and so on [32]. As the mostly studied target of miR-132/-212, SIRT1 played an important role in the development of tumors, inflammatory diseases, neurodegenerative and some other diseases [33–35]. It is worth noting that more and more studies indicated that SIRT1 activity could inhibit NLRP3 inflammasome activation and subsequent pyroptosis [36]. Xin Chou et al verified that overexpression of SIRT1 significantly abolished the activation of Cd-induced IRE-1α/XBP-1s axis, and then suppressed NRLP3 inflammasome activation as well as pyroptosis [37]. In the study, we also found that overexpression of SIRT1 can inhibit the expression of NLRP3 and the activation of Caspase-1, and could reverse the level of pyroptosis caused by LPS, suggesting that LPS can facilitate the pyroptosis pathway of NLRP3-Caspase-1 inflammasome by suppressing SIRT1.

**Figure 6:**
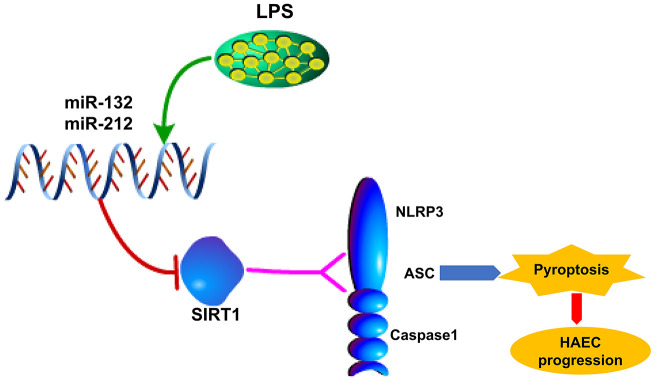
**Possible mechanism by which LPS upregulates miR-132/-212 in Hirschsprung-associated enterocolitis, facilitating pyroptosis by activating NLRP3 inflammasome via targeting Sirtuin 1 (SIRT1).**

Thus, we explored the role of LPS/miR-132/-212-SIRT1-NLRP3 regulatory network in HAEC by experiments in vitro carried out in LPS-injured HT29 cell line. The data presented in this study showed that LPS-induced upregulation of miR-132/-212, triggered activation of NLRP3 inflammasome and succedent pyroptosis in patients with postoperative HAEC and LPS-stimulated HT29 cell line and LPS-treated mice. Furthermore, miR-132/-212 inhibitor and SIRT1 overexpression transfection moderated LPS-induced cell pyroptosis (Figure 6). However, the above effect was not enough for abrogating cell pyroptosis, supporting the notion that other types of cell injury mechanisms may also exist. Summarily, our study provides a mechanistic explanation of LPS/miR-132/-212/SIRT1/NLRP3-Caspase-1 inflammasome in the progression of HAEC: LPS induces upregulation of miR-132/-212, which triggers Caspase-1-dependent pyroptosis via inhibiting SIRT1. It also proposes a new molecular pathway for LPS-mediated cell pyroptosis, which provides new insights into understanding the molecular mechanism of cell pyroptosis.

## MATERIALS AND METHODS

### Study population

From 2010 to 2015, patients were enrolled by the Children’s Hospital of Nanjing Medical University (Nanjing, Jiangsu Province, China). The ethical approval was obtained from the Institutional Ethics Committee of Nanjing Medical University (FWA00001501). This study cohort comprised 64 patients who were diagnosed with HSCR based on pathology and a half got postoperative HAEC. Written informed consent was obtained for each participant. Considering the influence of antibiotics to microbiome composition in preoperative HAEC patients, we chose HSCR patients with postoperative HAEC to explore the role of LPS in the occurrence of HAEC. The diagnosis of HAEC was in accordance with the guidelines established by Pastor AC et al [38]. All HSCR patients were followed up for 3 years after surgery to clear the occurrence of postoperative HAEC. All the narrow and dilated segments of guts were respectively collected and stored at -80°C immediately until use. Pathological analysis for excision of narrow segment was key for diagnosing HSCR. The dilated segment was applied to study for HAEC.

### Experimental animals and sample collection

C57BL/6 mice (20 ± 2 g, 6-8 weeks old) were obtained from SIPEIFU biotechnology co. LTD (Beijing, China). Approval for this study was received from the Animal Care and Use Committee in Nanjing Medical University and animal care was conducted according to guidelines of the Nanjing Ethics Committee. LPS (E. coli O111: B4; lot: 56H4096, Sigma) obtained via phenol extraction from serotypes were used. Mice were kept at the environment with stable temperature ranging from 24 to 28 °C with a 12 h light/dark cycle. The mice were randomly divided into two groups (n= 6 each): control and LPS groups. Control animals were injected normal saline intraperitoneally while LPS-treated mice received intraperitoneal (i.p.) injection of 10mg/kg LPS. After 24h LPS challenge, each mouse was sacrificed by decapitation and the intestinal tissues were obtained [39]. During experiments, the investigators were blinded to control and LPS groups.

### RNA isolation and qRT-PCR

We used TRIzol reagent (Life Technologies, CA, US) to extract tissue and cell RNA. Reverse Transcription Kit (Takara, Tokyo, Japan) did duty for obtaining cDNA. qRT-PCR reactions (ChamQ SYBR qPCR Master Mix, Vazyme) were carried out to measure the expression of miRNAs and mRNAs using Light Cycler 480 (Roche, Switzerland). The relative gene expression level was calculated by the 2^−ΔCt^ method. Fold change was normalized to internal reference gene GAPDH. As listed in Supplementary Table 1, all primers were involved in this study.

### Cell culture and transfection

HT29 cells (a human colorectal adenocarcinoma cell line) were obtained from the cell bank of the Chinese Academy of Science (Shanghai, China). HT29 cells were harvested into Dulbecco’s modified Eagle’s medium (DMEM), added with 10% fetal bovine serum (FBS) and 1% antibiotic–antimicotic solution. The culture condition was kept at 37°C, 5% CO2. A maximum of 6 cell passages was used. For simulating inflammation reaction in cell models, HT29 cells were exposed to LPS ranging from 0 to 1000 ng/ml. MiR-132/-212 mimics and inhibitors (GenePharma, Shanghai, China) were applied to regulate miR-132 and miR-212 expression in HT29 cell line. SIRT1 overexpressing plasmids were designed and synthesized by Jikai Co (Shanghai, China). All the above transfection experiments were conducted using Lipofectamine 3000 reagent (Invitrogen, Carlsbad, CA, USA) according to the manufacturer's instructions. All assays were conducted three times independently.

### Enzyme-linked immunosorbent assay (ELISA)

Serum samples were collected from control and LPS-stimulated mice. Concentrations of IL-1β and IL-18 in mice serum were assayed by ELISA kit (Elabscience) following the manufacturer’s protocol.

### Western blot analysis

Total protein was extracted by RIPA buffer and the concentrations were determined by bicinchoninic acid (BCA) solution (Beyotime, Nantong, China). We then performed Western Blot on the basis of the standard process. This study included the following primary antibodies: anti-SIRT1 (Santa Cruz, USA,1:1000, mouse, Cat. No.:sc-74465), anti-NLRP3(Abcam, Cambridge, MA, USA, 1:1000, rabbit, Cat. No.: ab214185), anti-Caspase-1(Abcam, Cambridge, MA, USA, 1:1000, rabbit, Cat. No.: ab1872), anti-ASC (Bioss, Beijing, China, 1:1000, rabbit, Cat. No.: bs-6741R), anti-β-actin (Santa Cruz, USA, 1:1000, mouse, Cat. No.: sc-81178). Image J was used to quantify band intensities (National Institute of Health, Bethesda, MD).

### Dual luciferase assay

TargetScan, picTar and miRanda analysis revealed the 3ʹ-UTR of SIRT1 as a potential binding site of miR-132-3p and miR-212-3p. The wild-type (WT) SIRT1-3ʹ-UTR and mutant (Mut) SIRT1-3ʹ-UTR containing the putative binding sites for miR-132 and miR-212 were chemically synthesized and cloned into the GV272 Dual-luciferase vector (Jikai, Hanghai, China). HT29 cells were seeded into 24-well plates and then transfected with a mixture of GV272 -Mut- SIRT1-3ʹ-UTR or GV272-WT- SIRT1-3ʹ-UTR and miR-132/-212 NC or miR-132/-212 mimics. After incubation for 48 h, the luciferase activity was measured by the dual Gloluciferase assay system (Promega, Madison, WI, USA).

### Immunofluorescence

Immunofluorescent labeling was used for assessing the expression and localization of SIRT1 and NLRP3 in HT29 cells. Firstly, the transfected cells were fixed with 4% paraformaldehyde for 30min, followed by blocked with goat serum. Subsequently, anti-SIRT1 (Santa Cruz, USA, 1:1000, mouse, Cat. No.: sc-74465) and anti-NLRP3 (Abcam, Cambridge, MA, USA, 1:1000, rabbit, Cat. No.: ab4207) antibody were diluted 1:200 to incubate cells at 4°C overnight. After incubated with FITC and Cy3 secondary antibody (Beyotime, Nantong, China) in the dark for 30 min, 4′,6-diamidino-2-pheny- lindole (DAPI; Beyotime, China) was applied to stain the nuclei for 15min. The cells were imaged with ×200 magnification under zeiss Laser Confocal Microscope. All assays were repeated 3 times independently.

### Flow cytometric analysis

FAM-FLICA in vitro Caspase-1 Detection Kit (ImmunoChemistry Technologie, Bloomington, MN, USA) was used for assessing Caspase-1 ability and cell pyroptosis. The fluorescence intensity of cells labeled with FAM-FLICA can be quantified using a flow cytometer. The specific procedure was implemented following the manufacturer’s protocol. In brief, we collected transfected cells and then incubated cells with 300μL cell culture media containing 10 μL FAM-FLICA at 37° 30min~1h. Next, cells were washed twice and read on a flow cytometer. In the diagram, ordinate represents cell count while the abscissa represents fluorescence intensity. The above diagram exhibited cell count on each fluorescence intensity and the mean value of cell Caspase-1 fluorescence intensity. The experiment was repeated 3 times.

### Data analysis

GraphPad Prism software was used for processing statistical analyses. In addition, Student’s t-test served for comparing two groups and the data were expressed as the mean±SEM. The Pearson’s correlation analysis was carried out to determine the coefficients of correlation (r). P<0.05 was considered statistically significant.

## Supplementary Material

Supplementary Figure 1

Supplementary Table 1
