# Supplementary material for: Lipopolysaccharide upregulates miR-132/212 in Hirschsprung-associated enterocolitis, facilitating pyroptosis by activating NLRP3 inflammasome via targeting Sirtuin 1 (SIRT1)
Source: Aging (Albany NY). 2020 Sep 20;12(18):18588–602. doi: 10.18632/aging.103852 (PMC7585123; doi:10.18632/aging.103852)
Supplement: Supplementary Figure 1 [file aging-12-103852-s002..pdf]

SUPPLEMENTARY FIGURE

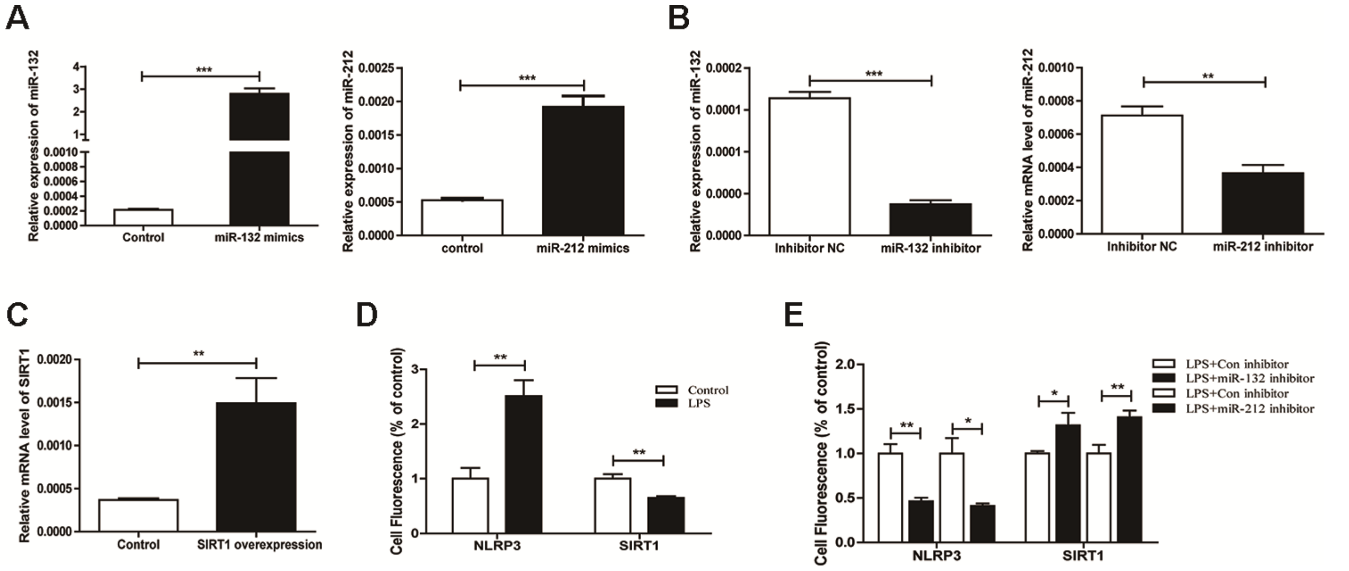

Supplementary Figure 1. (**A** and **B**) The miRNA levels of miR-132 and miR-212 were detected by qRT-PCR after HT29 cell line was transfected with miR-132/-212 mimics or inhibitors. (**C**) SIRT1 mRNA level in HT29 cell line was detected by qRT-PCR after treated with SIRT1 overexpression. (**D** and **E**) Cell fluorescence quantitative analysis of NLRP3 and SIRT1 showed high staining intensity of NLRP3 and inverse trend of SIRT1 in HT29 cell line treated with 1000ng/ml LPS, which could be reversed by miR-132/-212 inhibitors.
