# Supplementary material for: Lipopolysaccharide upregulates miR-132/212 in Hirschsprung-associated enterocolitis, facilitating pyroptosis by activating NLRP3 inflammasome via targeting Sirtuin 1 (SIRT1)
Source: Aging (Albany NY). 2020 Sep 20;12(18):18588–602. doi: 10.18632/aging.103852 (PMC7585123; doi:10.18632/aging.103852)
Supplement: Supplementary Table 1 [file aging-12-103852-s001..pdf]

## SUPPLEMENTARY TABLE

**Supplementary Table 1. Sequences of primers for qRT-PCR and siRNA related sequence.**

| | |
|---|---|
| SIRT1 | F: 5'-TGTGTCATAGGTTAGGTGGTGA-3' |
| | R: 5'-AGCCAATTCTTTTTGTGTTCGTG-3' |
| NLRP3 | F: 5'-CCACAAGATCGTGAGAAAACCC-3' |
| | R: 5'-CGGTCCTATGTGCTCGTCA-3' |
| Caspase-1 | F: 5'-TTTCCGCAAGGTTCGATTTTCA-3' |
| | R: 5'-GGCATCTGCGCTCTACCATC-3' |
| ASC | F: 5'-TGGATGCTCTGTACGGGAAG-3' |
| | R: 5'-CCAGGCTGGTGTGAAACTGAA-3' |
| GAPDH | F: 5'-GCACCGTCAAGGCTGAGAAC-3' |
| | R: 5'-GGATCTCGCTCCTGGAAGATG-3' |
| mmu-miR-132 | F: 5'-ACACTCCAGCTGGGTAACAGTCTACAGCCA-3' |
| | R: 5'-CTCAACTGGTGTCGTGGAGTCGGCAATTCAGT TGAGCGACCATG-3' |
| mmu-miR-212 | F: 5'- ACACTCCAGCTGGGTAACAGTCTCCAGTCA-3' |
| | R: 5'- CTCAACTGGTGTCGTGGAGTCGGCAATTCAGTT GAGTGGCCGTG-3' |
| mmu-SIRT1 | F: 5'- TGATTGGCACCGATCCTCG-3' |
| | R: 5'- CCACAGCGTCATATCATCCAG-3' |
| mmu-NLRP3 | F: 5'- ATTACCCGCCCGAGAAAGG-3' |
| | R: 5'- TCGCAGCAAAGATCCACACAG-3' |
| mmu-Caspase-1 | F: 5'- CTTGGAGACATCCTGTCAGGG-3' |
| | R: 5'- AGTCACAAGACCAGGCATATTCT-3' |
| mmu-ASC | F: 5'- CTTGTCAGGGGATGAACTCAAAA-3' |
| | R: 5'- GCCATACGACTCCAGATAGTAGC-3' |
| mmu-GAPDH | F: 5'- AGGTCGGTGTGAACGGATTTG-3' |
| | R: 5'- TGTAGACCATGTAGTTGAGGTCA-3' |
| miR-132 mimics | Sense: 5'-UAACAGUCUACAGCCAUGGUCG-3' |
| | Antisense: 5'-ACCAUGGCUGUAGACUGUUAUU-3' |
| miR-132 inhibitor | Sense: 5'-CGACCAUGGCUGUAGACUGUUA-3' |
| miR-212 mimics | Sense: 5'- UAACAGUCUCCAGUCACGGCC-3' |
| | Antisense: 5'- CCGUGACUGGAGACUGUUAUU-3' |
| miR-212 inhibitor | Sense: 5'- GGCCGUGACUGGAGACUGUUA-3' |
